# Land Use Zoning at the County Level Based on a Multi-Objective Particle Swarm Optimization Algorithm: A Case Study from Yicheng, China

**DOI:** 10.3390/ijerph9082801

**Published:** 2012-08-06

**Authors:** Yaolin Liu, Hua Wang, Yingli Ji, Zhongqiu Liu, Xiang Zhao

**Affiliations:** 1 School of Resource and Environmental Science, Wuhan University, Luoyu Road 129, Wuhan 430079, China; Email: whuwanghua@163.com (H.W.); zoneq6@163.com (Z.L.); zhaoxiang@whu.edu.cn (X.Z.); 2 Key Laboratory of Geographical Information System, Ministry of Education, Wuhan University, Luoyu Road 129, Wuhan 430079, China; 3 Department of Computer Science, Central China Normal University, Luoyu Road 152, Wuhan 430079, China; Email: lucky201199@163.com

**Keywords:** land-use zoning, multi-objective optimization, particle swarm optimization, crossover and mutation, Yicheng

## Abstract

Comprehensive land-use planning (CLUP) at the county level in China must include land-use zoning. This is specifically stipulated by the China Land Management Law and aims to achieve strict control on the usages of land. The land-use zoning problem is treated as a multi-objective optimization problem (MOOP) in this article, which is different from the traditional treatment. A particle swarm optimization (PSO) based model is applied to the problem and is developed to maximize the attribute differences between land-use zones, the spatial compactness, the degree of spatial harmony and the ecological benefits of the land-use zones. This is subject to some constraints such as: the quantity limitations for varying land-use zones, regulations assigning land units to a certain land-use zone, and the stipulation of a minimum parcel area in a land-use zoning map. In addition, a crossover and mutation operator from a genetic algorithm is adopted to avoid the prematurity of PSO. The results obtained for Yicheng, a county in central China, using different objective weighting schemes, are compared and suggest that: (1) the fundamental demand for attribute difference between land-use zones leads to a mass of fragmentary land-use zones; (2) the spatial pattern of land-use zones is remarkably optimized when a weight is given to the sub-objectives of spatial compactness and the degree of spatial harmony, simultaneously, with a reduction of attribute difference between land-use zones; (3) when a weight is given to the sub-objective of ecological benefits of the land-use zones, the ecological benefits get a slight increase also at the expense of a reduction in attribute difference between land-use zones; (4) the pursuit of spatial harmony or spatial compactness may have a negative effect on each other; (5) an increase in the ecological benefits may improve the spatial compactness and spatial harmony of the land-use zones; (6) adjusting the weights assigned to each sub-objective can generate a corresponding optimal solution, with a different quantity structure and spatial pattern to satisfy the preference of the different decision makers; (7) the model proposed in this paper is capable of handling the land-use zoning problem, and the crossover and mutation operator can improve the performance of the model, but, nevertheless, leads to increased time consumption.

## 1. Introduction

Land-use zoning and regulation, which originated in the late 19th century and was ubiquitous in most major US cities from the 1920s, is an effective measure for optimizing the allocation of land resources [1,2] and mitigating the negative externalities caused by mixed land use, and has been implemented in many countries and regions [3,4,5]. As the most important method of land-use regulation undertaken by local governments, zoning divides an administrative division into geographically contiguous ‘zones’, where each zone has its ordinance which prescribes what may and what may not be done [6]. The structure and goals of land-use regulation vary with the economic development, social institution and traditional culture of the different countries. China, since the adoption of economic reform and the open-door policy in 1978, has experienced rapid urbanization and industrialization, with the resulting undesirable phenomena of urban sprawl and expansion, loss of farmland, damage to the ecological environment, *etc*. Therefore, the main purpose of the zoning policy of China is to restrict the urban encroachment on rural lands, slow the agricultural conversion process and provide protection for the preservation of farmland and forest land [7]. Xinji County of Hebei Province, as a pilot county, adopted the nation’s first comprehensive zoning ordinance in 1987, but there was no specific legal provision for zoning until 1998 when Article 20 of the China Land Management Law introduced a regulation bringing in CLUP at the county level, which defines the land-use zones and the dominant land use of each zone. It can be seen, however, that the actual implementation of zoning policy occurred later in China, and the methods and laws for zoning and land-use regulation, in line with China’s actual land-use conditions, are still in the process of being explored. So, the focus of this paper is to construct a rational model serving land-use zoning in China, in view of the fact that zoning is the centerpiece of local planning and land-use regulations [8].

Standards for CLUP at the county level note clearly that, in general, a county can be divided into eight different zones comprising basic farmland preservation areas (BFPA), general agricultural land areas (GALA), forestry land areas (FLA), animal agricultural land areas (AGLA), construction land for towns and villages (CLTV), independent industrial and mining land areas (IIMLA), tourism land areas (TLA), and natural and humanistic landscape preservation areas (NHLPA). Each land-use patch should be assigned to a certain zone, based on the actual land-use vector map [9,10]. A county can improve the land-use multi-efficiency and achieve the optimal allocation and sustainable utilization of land resources by strictly controlling the usages of land in each zone [11]. Traditional methods such as clustering analysis [12], the aggregative indicator method [13], and principal component analysis [14] are used for land-use zoning. All these methods are conducted based on the natural and socio-economic attributes of the land-use patches. However, land-use zoning is not only a spatial cluster problem, but also a multi-objective optimization problem, with the best contiguity between units belonging to the same zone and the highest economic returns, social effects and ecological benefits of land use [15]. Therefore, the aforementioned methods have obvious drawbacks in coping with land-use zoning with a MOOP. Furthermore, up to now, there's few research on solving land-use zoning problems with multi-objective optimization techniques.

Nevertheless, many multi-objective optimization models have been presented for solving the optimization of land-use structure and land-use allocation, which are also the significant components of land-use planning as land-use zoning in China. For example, Brookes [16] used a genetic algorithm (GA) to find the best configuration for patches subject to multiple criteria; Aerts [17] adopted linear integer programming (LIP) for multi-site land-use allocation, to minimize development costs and maximize compactness; Liu *et al*. [18] analyzed the application of a GA in the optimization of land-use structure, considering the economic benefits, ecological ‘green’ equivalent, and soil erosion; Santé *et al*. [19] applied simulated annealing (SA) in rural land-use allocation, to reconcile multiple conflicting interests such as land suitability and compactness; Sadeghi *et al*. [20] optimized land resource allocation by using linear programming, to minimize soil erosion and maximize economic returns; Gao *et al*. [21] generated an optimized land-use structure which is able to reduce soil erosion, enhance the utilization of water resources and raise agricultural productivity, based on a multi-objective programming (MOP) model and the geographic information system (GIS) technique; Eldrandaly [22] indicated that integrating GIS and gene expression programming (GEP) is a promising and efficient approach for solving multi-site land-use allocation (MLUA) problems; and Zhang *et al*. [23] applied a multi-agent system (MAS) to regional land-use optimization allocation under a multi-objective constraint. Multiple objectives defined in a spatial context leads to a complex spatial optimization problem and often exhibits substantial computational complexity, which makes exact optimization methods such as LIP infeasible when there are a large number of land units [17,24]; however, optimization techniques that incorporate heuristics have been found to be more effective in solving spatial optimization problems such as SA, GA [25].

Therefore, why not build a multi-objective optimization model for land-use zoning according to previous studies? The particle swarm optimization (PSO) method put forward for the first time by Kennedy and Eberhart [26], is a relatively recent heuristic search method whose mechanics are inspired by the swarming or collaborative behavior of biological populations, which is known as swarm intelligence [26]. As an evolutionary algorithm, it is based on a population of candidate solutions (swarm of particles), which have improved capability for solving complex problems, high convergence speed and good generality for different global optimization problems [27]. The most important advantages of PSO, compared to other optimization strategies, are that PSO is easy to implement and there are few parameters to adjust [28]. It therefore has wide applications in solving the MOPs in many fields [29,30,31,32].

Yicheng, a county-level city in Hubei Province, with rapid economic growth and a sensitive ecological environment, was chosen as the study area in this study. PSO was applied to the land-use zoning of Yicheng, employing an objective function that took into account the differences between zones, the spatial compactness of zones, the degree of spatial harmony of land-use zones, as well as the ecological benefits of land-use. The model obtained the Pareto-optimal solutions, with trade-off between the conflicting objectives, and was subject to the three typical constraints of containing a minimum parcel area in the land-use zoning map, limiting the area of land-use zones, and the general rules of dividing land units into various land-use zones. Additionally, considering the shortcomings of premature convergence and possibility of sinking into local optima of a basic PSO algorithm, a constriction factor and crossover and mutation operator were used in this model [33,34]. The algorithm, with several different sets of weights applied to these four objectives, was run, and the corresponding results were compared.

The remainder of the paper is organized as follows: Section 2 gives an introduction to the study area and relevant data. The application of PSO in a land-use zoning problem is described in Section 3. Thereafter, in Section 4, the solutions with different sets of weights are compared, and the improvements are discussed. Finally, the main conclusions are given in Section 5. 

## 2. Data

### 2.1. Study Area

**Figure 1:**
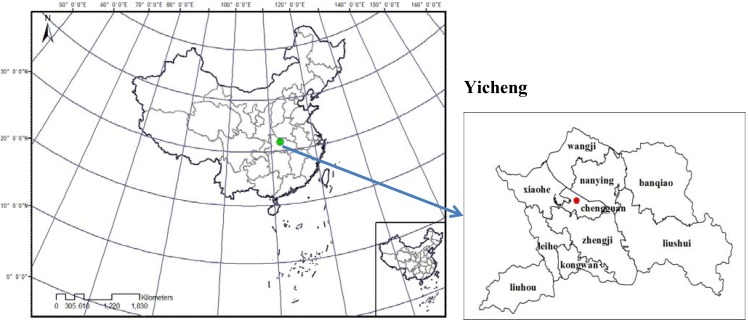
Location of the study area.

The study area (31°26′–31°54′N, 111°57′–112°57′E) is located in Hubei Province, central China, with an area of 2,114.75 km^2^ (Figure 1). The area, whose average elevation is between 50 and 150 m, accounts for 76.8% of the total county area, and a dense river network and many ponds cover the whole area. The average annual rainfall in this area, which has a typical subtropical monsoon climate, is between 800 and 1,000 mm. For this area, the population was 5.15 × 10^5^ and the average annual growth rate of total gross domestic product (GDP) in the 11th five-year period was more than 10%. The study area has abundant mineral resources, including silica, limestone, and the biggest deposits of bauxite in Hubei Province. Extensive industrial development of the area has led to a great threat to the local ecological environment.

In accordance with the CLUP (2006–2020) for Yicheng, the land-use zone of CLTV should continue to be divided into two zones comprising the construction land for towns (CLT) and construction land for villages (CLV). In addition, because there is little grassland for animal agriculture in this study area, the zone of LLA is excluded. In the end, the scenario of land-use zoning for Yicheng makes sure that eight land-use zones, including BFPA, GALA, FLA, CLV, CLT, IIMLA, TLA and NHLPA, are obtained.

### 2.2. Data Collection and Processing

The natural and social characteristics of each land-use patch should be considered when the work of land-use zoning is conducted, so we chose some indices that can reflect these characteristics and list them in Table 1.

**Table 1:** Land-use zoning indices.

Target	Criteria	Indices
Land-use zoning	soil quality	surface soil type, soil organic matter content, pH value,
		soil depth, soil erosion intensity, soil profile construction
	topographic	elevation, slope
	infrastructure	irrigation guarantee rate,
	for farmland	drainage conditions, rural road density
	land location	distance from rural settlements,
		distance to center of towns, distance from roads
	land-use status	land-use type

Land-use type can be quantized as an integer number such as 1, 2…n.

The soil quality data such as pH value, soil depth, soil erosion intensity, *etc*., were obtained from the soil quality survey map (1:50,000). Slope and elevation were derived from a digital elevation model (DEM of 30 m resolution, provided by the International Scientific & Technical Data Mirror Site, Computer Network Information Center, Chinese Academy of Sciences). Some vector traffic maps (1:10,000) were used to provide the spatial information such as centers of towns, railways, highways, roads, and administrative boundaries (provided by local bureau of land and resources). The type of each land-use patch, the percentage area of each land-use type and the distribution of rural settlements were obtained from the actual vector land-use map (1:10,000, provided by the local Bureau of Land and Resources). The actual land-use map contains 47,851 patches, with each patch considered to be a land unit which should be assigned to a certain land-use zone. Several fields were added to the actual land-use map to store the values of the aforementioned indices, after being standardized by Equation (1):


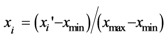
(1)

where *x_i_*’ is the value of variable *i* before standardization, *x_i_* is the value after standardization, *x_min_* is the minimum value of the variable, and *x_max_* is the maximum.

**Figure 2:**
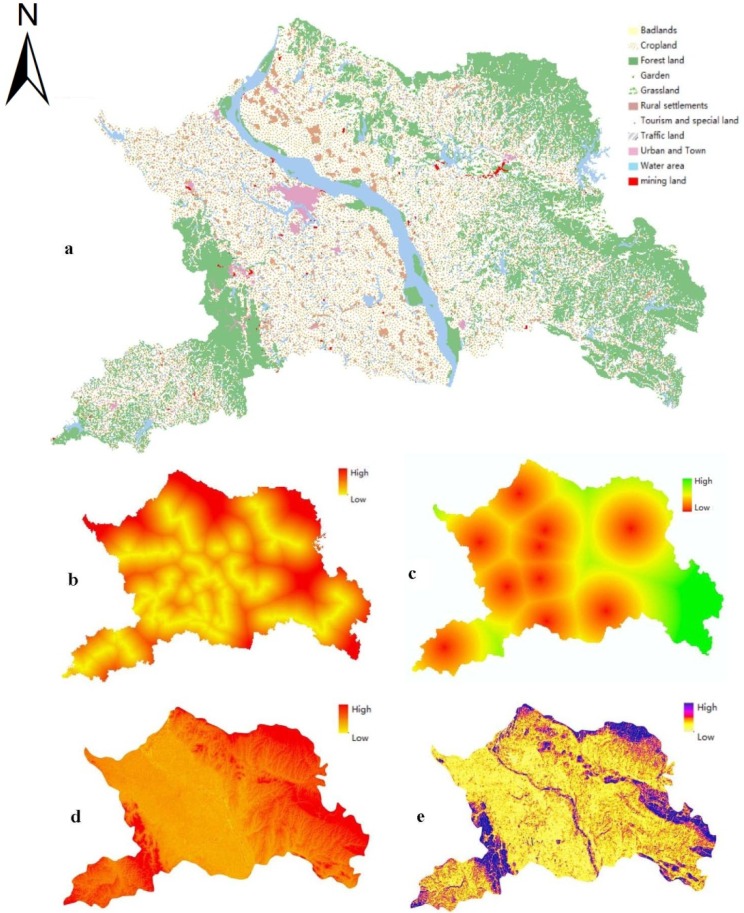
Main spatial data for the land-use zoning model: (**a**) Actual land-use map; (**b**) Distance from highways and railways; (**c**) Distance to center of towns; (**d**) Elevation; (**e**) Slope.

All the spatial data were processed and computed by the spatial analysis tool in ArcGIS 9.3. To ensure consistency of the data, all the images and maps were geometrically rectified with each other and subsequently referenced to the Gauss-Kruger projection Xian_1980_3_ Degree_GK_Zone_37 Projected Coordinate System. Figure 2 represents the main spatial data after GIS processing.

Land-use planning based on ecological evaluation is of great significance to sustainable development [35]. The ecological benefits of land use can be evaluated by the ecosystem services value (ESV) [36], where different land-use types and zones have different ESVs. Xie *et al*. [37] established the ESV unit areas of Chinese terrestrial ecosystems, based on the partial global ESV evaluation results obtained by Costanza *et al*. [38]. This paper defines the level of ecological benefit and the corresponding coefficient of different land-use zones (see Table 2) in accordance with the foregoing results, integrated with the responses to ecological questionnaires from specialists of different departments in Yicheng City.

**Table 2:** The ecological benefit level and coefficients of different land-use zones.

Land-use zone	NHLPA	FLA	GALA	TLA	BFPA	CLV	CLT	IIMLA
Level	1	2	3	4	5	6	7	8
Coefficient	1	0.9	0.6	0.5	0.4	0.3	0.1	0

## 3. Methodology

### 3.1. Objective Function

Optimized land-use zones can effectively improve the land-use multi-efficiency, achieve optimal allocation and guarantee sustainable utilization of land resources; therefore, each land-use patch should be assigned to a certain zone, not only considering the natural and social characteristics of each land-use patch but also the global spatial compactness, spatial harmony of the land-use zones and the ecological benefits of land use. Hence, optimal land-use zones should reach the following objectives:

(1) The attribute difference between land-use zones max*f*_AD_

Land-use zoning needs to divide n patches *x^d^*(*d* = 1,2,…,m, m is the number of attributes) into C land-use zones *G_j_*(*j* = 1,2,…c). The difference between all the land-use zones is greatest when the sum of the distances from each land-use patch to the center of the corresponding land-use zone is smallest. So, the problem can be formulated as:


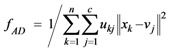
(2)

where n is the number of land-use patches and *c* is the number of land-use zones when patch *x_k_* belongs tozone *G_j_*, then *u_kj_* is equal to 1, or otherwise equal to 0. *v_j_* is the center of zone *G_j_* that can be calculated by formula 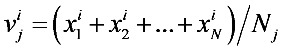, 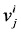
is the value of attribute *i* for the center of zone *G_j_*, 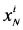
is the value of attribute *i* for patch *x_N_* that belongs to zone *G_j_*, *N_j_* is the total number of patches that belongs to zone *G_j_*, and 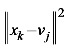
is the Euclidean distance between patch *x_k_* and *v_j_* that is given by formula 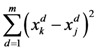.

(2) The spatial compactness of land-use zones max*f*_SC_

To meet the needs of land-use control, besides the optimization of the quantity and quality of land use, the optimization of the spatial pattern is also an important component of land-use zoning. Reducing the fragmentary zones benefits the spatial control. The problem can be formulated as Equation (3):


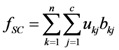
(3)

**Figure 3:**
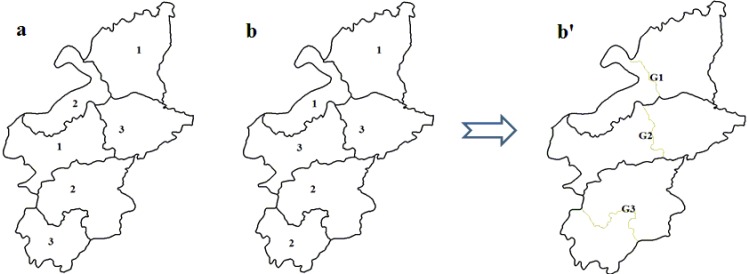
The spatial pattern of land-use zoning: the spatial compactness of **b** is obviously better than **a**; **b′** represents the spatial clusters.

If land-use patch *x_k_* is assigned to spatial cluster *G_j_*, *b_kj_* is the number of adjacent patches that also belong to *G_j_*. *f_SC_* achieves a maximum value when land-use patches with the same attributes are adjacent to each other, and *f_SC_* achieves a minimum value when each patch has no adjacent patches that belong to the same zone, a small area with several patches in Yicheng is chosen to describe this vividly (Figure 3).

(3) The degree of spatial harmony of land-use zones max*f*_HD_

While in the process of partitioning the n patches into C land-use zones and D spatial clusters (Figure 3b), the state and influence of the neighborhood of each zone (spatial cluster) should be considered. The proper adjacent distribution of two land-use zones will promote the convenience degree of social production activities of human. Therefore, the problem can be expressed as in Equation (4):


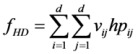
(4)

where *d* is the number of spatial clusters, *v_ij_* is equal to 1 when cluster *i* and cluster *j* are adjacent to each other, otherwise *v_ij_* is equal to 0. *hp_ij_* represents the spatial harmony index between cluster *i* and cluster *j*, which belong to two different zones, respectively. The spatial harmony index between different land-use zones is obtained according to a combination of expert experience and the Delphi method, the results are listed in Table 3.

The ecological benefits of land-use zones max*f*_EB_: Different land-use patterns provide a different ESV. Objective *f*_EB_ aims to improve the global ESV, based on the optimal land-use zones from an ecological point of view. So, the problem can be expressed as follows:


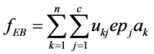
(5)

where *ep*_k_ represents the ecological benefit coefficient (refer to Table 2) when patch *x_k_* is allotted to zone*j*. *f_EB_* achieves a maximum value when all the land-use patches are assigned to the zone of FLA, and *f_EB_* achieves a minimum value when all the patches are assigned to the zone of CLTV or IIMLA.

**Table 3:** The spatial harmony index between different land-use zones.

Land-use zone	BFPA	GALA	FLA	CLV	CLT	IIMLA	TLA	NHLPA
BFPA	1	0.8	0.6	0.5	0.1	0.1	0.3	0.4
GALA		1	0.8	0.5	0.2	0.1	0.3	0.4
FLA			1	0.3	0.3	0.3	0.8	0.8
CLV				1	0.8	0.6	0.4	0.2
CLT					1	0.3	0.2	0.1
IIMLA						1	0.1	0.1
TLA							1	0.6
NHLPA								1

Since these four objective functions (Equations (2–5)) represent conflicting land use demands, the scenario of land-use zoning with the optimal spatial pattern does not promise to give the maximum ecological benefits; therefore, there does not exist a single ideal solution which simultaneously satisfies the decision makers across all criteria. So, a compromise solution is sought based on a linear weighting method, which is a simple and practical way to convert the multi-objective problem into a single-objective one by using the following expression:


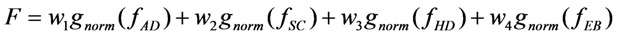
(6)

where *w_1_*, *w_2_*, *w_3_* and *w_4_* are the weights of four objective functions, respectively, and subject to the condition *w_1 _*+ *w_2 _*+ *w_3_* +*w_4_* = 1. *g_norm_* normalizes the values of the objectives within the range [0, 1] to eliminate the impact of different dimensions by:


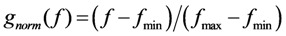
(7)

### 3.2. Constraint Condition

#### 3.2.1. The stipulation of a minimum parcel area in a land-use zoning map

The standards for CLUP at the county level make clear a regulation about the minimum parcel area (MPA) of different land-use zone types in the land-use zoning map (see Table 4). The parcels (also spatial clusters) with a conflict with the regulation should be merged into an adjacent parcel which has a maximal area.

**Table 4:** The MPA in a land-use zoning map with 1:10,000 scale.

Land-use zone	BFPA	GALA	FLA	CLV	CLT	IIMLA	TLA	NHLPA
Area in map (mm^2^)	20	20	100	20	20	20	20	100
Actual area (hm^2^)	0.2	0.2	1	0.2	0.2	0.2	0.2	1

#### 3.2.2. The limitation of area of various land-use zones

In the process of land-use zoning, the quantity of land-use zones should meet the demands of the land-use plan, as well as the optimization of the spatial pattern. In accordance with the CLUP (2006–2020) for Yicheng, which states that the area of basic farmland should be not less than 62,462.14 hm^2^ by 2020, and the amount of total construction land should not exceed 18,785.70 hm^2^, the forest coverage rate will simultaneously have to reach 26.36% (55,563.16 hm^2^), so the area of BFPA, FLA, CLV, CLT and IIMLA should satisfy the inequality as follows:


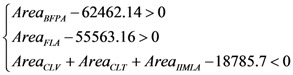


#### 3.2.3. The general regulations of land-use zoning

**Figure 4:**
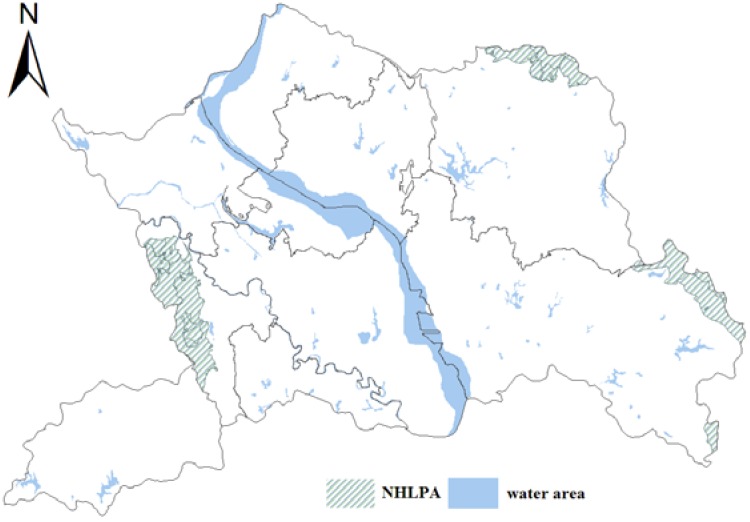
Water and nature reserve areas in Yicheng.

(1) Land-use zoning is conducted excluding the water areas. Therefore, the 527 land-use patches for the water areas will not participate in the land-use zoning and will be given a value of NONE in the attribute of land-use zone. The total area of water areas in the study area is 12,998.06 hm^2^ (see Figure 4).

(2) Nature reserve areas with important ecological value, special cultural relics, historical sites and ecological forest must be assigned to NHLPA as a priority, and the attribute values of 112 land-use patches for the above types remain to the very end of the process of land-use zoning, with an area of 10,111.58 hm^2^ (see Figure 4).

(3) There is little likelihood of built-up areas being converted into other land-use types, based on the general laws of land-use change [39,40], so the land-use patches for urban areas and towns, and industry and mining should be only assigned to CLT and IIMLA, respectively. The same applies for tourism land, which can only be assigned to TLA.

(4) Cheng *et al*. [41] defined the general dominant land use, allowable land use, and prohibited land use in various land-use zones. This paper also provides the relevant regulations for the eight land-use zones of Yicheng, in line with the local conditions (see Table 5). The probability interval of a land-use patch of a certain type being assigned to a zone is determined based on the information from Table 5, and is listed in Table 6. The zone label of each land-use patch should be decided referring to the table of probability intervals, for the initialization of different scenarios of land-use zoning, as follows:


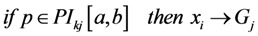
(8)

where p is a random number and 0 ≤ p ≤ 1, *PI_kj_*[*a,b*] is the probability interval of land-use type *k* being allocated to land-use zone type *j*.

**Table 5:** The dominant land use, allowable land use, and prohibited land use of the various land-use zones.

Land-use zone	BFPA	GALA	FLA	CLV	CLT	IIMLA	TLA
Cropland	△	□	□	―	―	―	―
Garden	□	△	□	―	―	―	□
Grassland	□	□	□	□	□	□	□
Forest land	□	□	△	□	□	□	□
Traffic land	□	□	□	□	□	□	□
Rural settlements	―	□	□	△	―	―	□
Tourism and special land	―	―	―	―	―	―	△
Badlands	―	―	―	□	□	□	□

△ dominant land use, □ allowable land use，― prohibited land use.

**Table 6:** The probability intervals of certain land-use types being allocated to certain land-use zone types.

Land-use zone	BFPA	GALA	FLA	CLV	CLT	IIMLA	TLA
Cropland	[0, 0.8]	[0.8, 0.9]	[0.9, 1]	―	―	―	―
Garden	[0.7, 0.85]	[0, 0.7]	[0.85, 0.95]	―	―	―	[0.95, 1]
Grassland	[0.6, 0.7]	[0.7, 0.8]	[0.8, 0.9]	[0.2, 0.4]	[0, 0.2]	[0.9, 1]	[0.4, 0.6]
Forest land	[0.7, 0.75]	[0.75, 0.8]	[0, 0.7]	[0.8, 0.85]	[0.85, 0.9]	[0.9, 0.95]	[0.95, 1]
Traffic land	[0.6, 0.7]	[0.4, 0.6]	[0.7, 0.8]	[0, 0.2]	[0.2, 0.4]	[0.8, 0.9]	[0.9, 1]
Rural settlements	―	[0.7, 0.8]	[0.8, 0.9]	[0, 0.7]	―	―	[0.9, 1]
Badlands	―	―	―	[0, 0.25]	[0.25, 0.5]	[0.5, 0.75]	[0.75, 1]

### 3.3. The Particle Swarm Optimization Model

As an important branch of swarm intelligence, the PSO algorithm maintains a population of particles, where each particle represents a potential solution to an optimization problem. The set of particles, also known as a swarm, is flown through the D-dimensional search space of the problem. The position of each particle is changed, based on the experiences of the particle itself and those of its neighbors [42]. The particle in the model of land-use zoning, based on the multi-objective particle swarm optimization with constriction factor, and crossover and mutation operator (MOPSO-CCM), is seen as a potential scenario of land-use zoning. The velocity and position of the particle are updated under the guidance and restriction of the aforementioned objective functions and constraints. To implement the application of MOPSO-CCM in land-use zoning, it is necessary to define the coding scheme for the particle, design the crossover and mutation operator, and set up the updating mechanism for the particle’s position and velocity, according to the particular land-use zoning problem.

**Figure 5:**
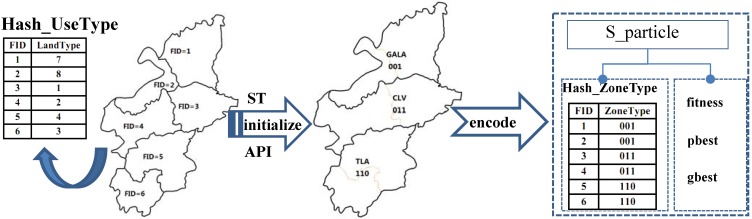
The coding scheme for a particle.

(1) Coding scheme of the particle (Figure 5)

The eight land-use zones are encoded with a hybrid code system consisting of both decimal code and binary code (see Table 7). Decimal code is defined for the updating of the particle’s position, and binary code is proposed for the crossover and mutation of the particle. The encoded information is stored in a relational table in the database, which is convenient for the operation of encoding and decoding.

**Table 7:** The coding of the land-use zones.

Land-use zone	BFPA	GALA	FLA	CLV	CLT	IIMLA	TLA	NHLPA
Decimal code	0	1	2	3	4	5	6	7
Binary code	000	001	010	011	100	101	110	111

The land-use patches are encoded with a serial integer and unique numbers in ascending order. The code of each land-use patch and corresponding land-use type are stored in a hash table named as Hash-UseType.

When the initialization of a population of random particles starts, each land-use patch obtains the type of land-use zone, based on the stochastic technique (ST) integrated with assigning the probability interval (API, refer to Table 6). The relevant information is stored in a hash table named as Hash-ZoneType.

The particle can be represented by the structure named as S_particle, which consists of a hash table (Hash-LandZoneType), the current fitness (fitness), the best fitness achieved by itself (pbest) and the best fitness obtained by any particle in the population (gbest). The array of S_particles is used to represent the swarm of particles.

**Figure 6:**
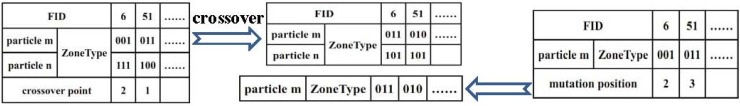
The crossover and mutation operator.

(2) Designing the crossover and mutation operator (Figure 6)

In the process of crossover or mutation, the particle will be seen as a chromosome, and a land-use patch will be seen as a gene.

A single-point crossover operator is adopted to reproduce offspring. The selection of parents for performing crossover is done at a specified probability and is displayed in the mating pool. The number and FID code of genes preparing for crossover are determined randomly. For the purpose of the crossover operation, the crossover point i (i∈{1,2,3}) is also chosen randomly, then the corresponding binary bits before and after the crossover point of the parents are interchanged, leading to two new individuals. 

The mutation operator is designed as follows. A chromosome is chosen for mutation at a preset probability. Some genes and the corresponding position i (i∈{1,2,3}) for mutation then need to be chosen randomly. Thanks to the gene structure of the binary code, if the value at the mutation position is 1, then reset it to 0, otherwise to 1.

When crossover or mutation is proceeding, the constraint of API should be considered. For example, cropland is prohibited in the land-use zone of TLA, so if the transition of the gene offends against the constraint of API, the operation of crossover or mutation should be repeated until the transition is acceptable or reaches a preset try-number.

(3) Updating mechanism of the particle’s position and velocity

A new position for the particle is computed by the following formula in the basic PSO with inertial weight [43].


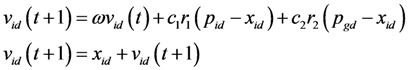
(9)

where *x_id_* and *v_id_* are the position and velocity of a particle, *r_1_* and *r_2_* are two distinct random values in [0, 1], *c_1_* and c*_2_* are acceleration constants, *p_id_* and *p_gd_* are the best previous position of the particle itself (pbest) and of all the particles of the swarm (gbest), respectively. 

Eberhart and Shi [44] found that constriction factor *k* introduced by Clerc [45], combined with constraints on *V_max_* (equal to *x_max_*), significantly improved the PSO performance. The formula used to compute the velocity is then modified as: 


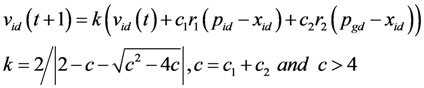
(10)

The land-use zoning problem can be regarded as an integer programming problem, which is described as follows:


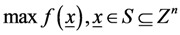


where Z^n^ is the n-dimensional integer space, and *S* is an integer set. In this paper, *S* can be represented as {x|0≤ x≤ 7 and x∈Z^n^}.

When PSO is used to solve the land-use zoning problem, the new position of the particle may be changed to a real number, even though the previous location and velocity are both integers, because the parameters of *k*, *r_1_*, *r_2_*, *c_1_* and *c_2_* are all real numbers. To make sure the particles are flown through the integer number space of the problem, *v_id_* (*t + 1*), including the three parts of *kv_id_* (*t*), *kc_1_r_1_* (*p_id_-x_id_*), and *kc_2_r_2_*(*p_gd_-x_id_*), must be an integer number in the evolutionary process. *kv_id_* (*t*) can be changed into an integer number by function int(). *kc_1_r_1_* (*p_id_-x_id_*) is defined as *μ_1_*, when *p_id _*> *x_id_*, and *μ_1_* is a random number within [0, kc1 (pid-xid)], otherwise within [-*kc_1_*(*p_id_-x_id_*),0], so we can also define *μ_1_* as a random number within [*a_1_*, *b_1_*] for simplification. On the assumption that there are n integer numbers in [*a_1_*, *b_1_*], any integer number can be chosen to set to *μ_1_*, with an equivalent probability equal to 1/n. *kc_2_r_2_*(*p_gd_-x_id_*) is defined as *μ_2_* with the same processing method. So, the formula to compute the velocity of the particle can be modified again as:


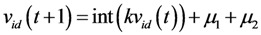
(11)

As with the crossover and mutation, it may also offend against the constraint of API when the position of the particle is updating. Simultaneously, *x_id_*(*t* + 1) of the particle may be outside the range of S. So, the attribute of the land-use patch will be randomly decided, subject to the API when the phenomenon mentioned above occurred.

**Figure 7:**
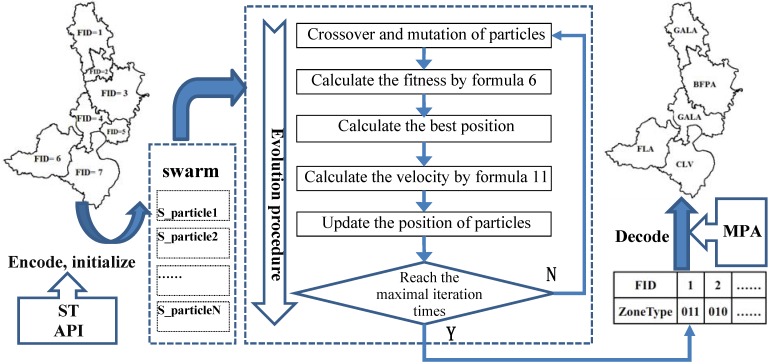
The crossover and mutation operator.

Figure 7 presents a flow diagram of the MOPSO-CCM model, which is based on the objective functions, constraint condition, encoding and decoding scheme, and updating mechanism for the swarm.

The performance of the model depends on several critical parameters [46], including the population size *N*, acceleration constants *c_1_* and *c_2_*, constriction factor k and maximum velocity *V_max_*. Carlisle and Doziert [47] put forward a classical combination of parameters, with *N* = 30, *c_1_* = 2.8, *c_2_* = 1.3 and *k* = 0.7299. Additionally, we set *V_max_* = 7, and the maximum number of iterations *Maxtimes* = 200.

## 4. Results and Discussion

### 4.1. Comparison of the Different Scenarios

The MOPSO-CCM model adopted the crossover and mutation operator by setting the probability of mutation and crossover to 0.3 and 0.01, respectively, and the try-number to 3, and afforded several scenarios of land-use zoning by using different sets of weights applied to the four sub-objectives. The weights *w_n_* given to the various objectives were obtained according to the sub-objective weighting schemes in the study of Santé *et al*. [19], and are listed in Table 8.

**Table 8:** Objective weighting schemes used in the MOPSO-CCM optimization.

Scenario	*w_1_*	*w_2_*	*w_3_*	*w_4_*
A	1	0	0	0
B	0.75	0.25	0	0
C	0.5	0.5	0	0
D	0.25	0.75	0	0
E	0.75	0	0.25	0
F	0.5	0	0.5	0
G	0.25	0	0.75	0
H	0.5	0	0	0.5
I	0.5	0.25	0.25	0
J	0.5	0.25	0	0.25
K	0.5	0	0.25	0.25
L	0.25	0.25	0.25	0.25
M	0.15	0.55	0.15	0.15
N	0.15	0.15	0.55	0.15
O	0.15	0.15	0.15	0.55

**Table 9:** The value of the four sub-objectives in the various weighted scenarios.

scenario	*f*_AD_	*f*_SC_	*f*_HD_	*f*_EB_
A(1/0/0/0)	0.0000524	189,563	13,632.8	104,486.6
B(0.75/0.25/0/0)	0.0000348	470,961	13,163.5	102,293.1
C(0.5/0.5/0/0)	0.0000335	487,165	12,836.3	101,294.5
D(0.25/0.75/0/0)	0.0000331	518,263	12,195.7	109,729.2
E(0.75/0/0.25/0)	0.0000448	178,954	18,875.9	101,195.4
F(0.5/0/0.5/0)	0.0000365	185,642	21,394.5	118,918.8
G(0.25/0/0.75/0)	0.0000342	169,637	23,163.2	110,073.9
H(0.5/0/0/0.5)	0.0000348	280,523	15,521.6	126,753.4
I(0.5/0.25/0.25/0)	0.0000339	365,651	17,915.5	115,095.3
J(0.5/0.25/0/0.25)	0.0000326	459,232	14,835.1	122,956.8
K(0.5/0/0.25/0.25)	0.0000345	238,632	19,933.7	123,346.1
L(0.25/0.25/0.25/0.25)	0.0000341	396,535	16,969.3	122,586.7
M(0.15/0.55/0.15/0.15)	0.0000329	445,689	16,289.6	121,366.5
N(0.15/0.15/0.55/0.15)	0.0000331	361,542	18,953.8	121,626.7
O(0.15/0.15/0.15/0.55)	0.0000325	402,549	17,386.4	124,054.2

The values of the four sub-objectives were obtained by MOPSO-CCM from the varied weighting scheme scenarios listed in Table 8 (see Table 9). The results in Table 9 show that whenever the objective of spatial compactness was included in the MOPSO-CCM objective function, along with the sub-objective of difference between land-use zones, the solution obtained displayed a significant expected increase ranging from 90.72% to 173.4% in *f*_SC_ and a reduction ranging from 33.59% to 37.98% in *f*_AD_, with comparison Scenarios B–D, I–J and L–O with Scenario A. The same can be seen for the degree of spatial harmony and ecological benefit sub-objectives, with respect to Scenario A, Scenarios E–G, I and K–O increase *f*_HD_ by 19.49% to 69.91%, in exchange for a reduction in *f*_AD_ of 14.5% to 37.98%, and Scenarios H and J–O get a slight increase in *f*_EB_ of 16.16% to 21.31%, at the expense of a reduction in *f*_AD_ of 33.59% to 37.98%.

**Figure 8:**
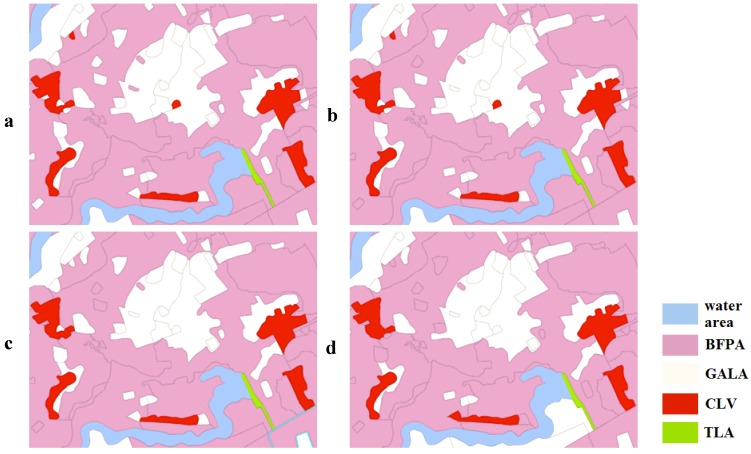
The effects of *w_2_* on optimizing the spatial pattern in different weighted scenarios: (**a**) A; (**b**) B; (**c**) C; (**d**) D.

When the objective function of the MOPSO-CCM model only included the sub-objective of spatial compactness (Scenarios B–D), the value of *f*_AD_ increased significantly by a factor of 2.5 as *w_2_* increased. The fundamental demand for attribute difference between land-use zones leads to a mass of fragmentary land-use zones; however, the sub-objective of spatial compactness can reduce the fragmentation by merging isolated land-use zones into a large contiguous land-use zone. For example, isolated CLV or GALA in a BFPA encourages merging into the BFPA. The garden land or rural settlements in BFPA will tend to be converted into farmland of high quality by consolidation of these lands in the future. Also, small BFPA or GALA showing up in a FLA encourages merging into the FLA, so the farmland with a slope greater than 25°, and a part of the farmland between 15° and 25°, should be transformed gradually into forest or grassland in the future. A typical small region is chosen from the villages of Qianfeng and Xinji to show how the sub-objective of spatial compactness optimizes the spatial pattern of land-use zones (see Figure 8).

**Figure 9:**
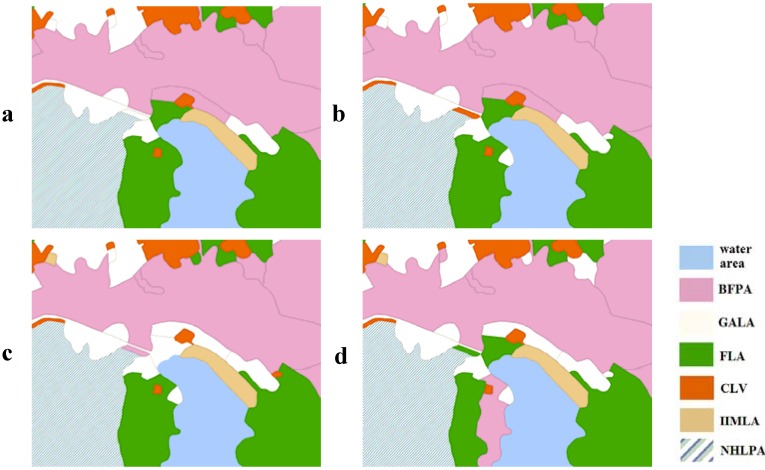
The effects of *w_3_* in improving the spatial harmony in different weighted scenarios: (**a**) A; (**b**) E; (**c**) F; (**d**) G.

When only the sub-objective of the degree of spatial harmony degree was included, f_HD_ had a gradual increase by a factor of more than 1.35 as w_3_ increased, which can be seen from Scenarios E–G. The improvement of spatial harmony requires that the spatial pattern of BFPA is adjacent to GALA, NHLPA is contiguous to FLA, but IIMLA should be distant from BFPA, *etc*. A small area chosen from the village of Quanshui is presented in Figure 9 to show how the suitable neighbors are allocated to a certain land-use zone as *w_3_* increases.

When *w_1_* is fixed as 0.5, the remainder of the weight is assigned equally to *w_2_* and *w_3_* (Scenario I), with a distinct decrease in *f*_SC_ compared with the remainder of the weight being all assigned to *w_2_* (Scenario C). When it is equally assigned to *w_2_* and *w_4_* (Scenario I), in spite of *f*_SC_, it still had a slight reduction compared to Scenario C, but it was obviously greater than Scenario I. This may suggest that the pursuit of spatial harmony will bring about more fragmented land-use zones, which means worse spatial compactness, which can also be seen from the comparison between Scenarios E–G and graphically in Figure 9. An increase in the ecological benefits can also improve the spatial compactness. From Scenarios A–D, we can see that *f*_HD_ reduced as *f*_SC_ increased, which proved that the spatial compactness also has a negative effect on spatial harmony. Another comparison of Scenario A with Scenarios H and J shows that *f*_HD_ increased by a factor of about 1.1 as *f*_EB_ increased, which suggests that the increase in ecological benefits will boost the degree of spatial harmony of the land-use zones.

**Figure 10:**
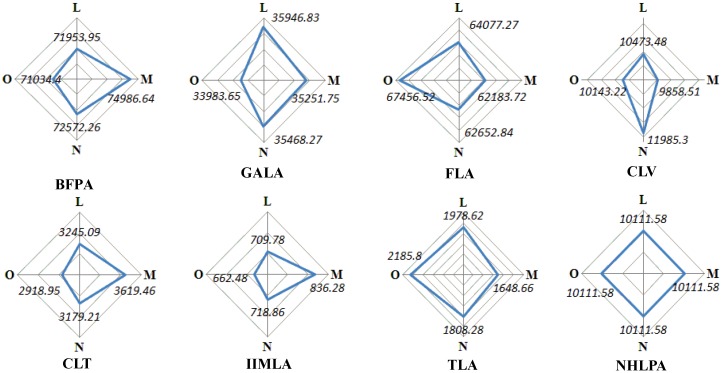
The area comparison of land-use zones between Scenarios L, M, N and O (hm^2^).

From the comparison of Scenario L with Scenarios M–O, it can be seen that assigning more weight to a certain sub-objective, and the same weight to other sub-objectives, had the expected effect of increasing the value of the corresponding sub-objective, indicating that the fitness values are positively correlated with the weights. That is to say, a decision maker for land-use planning can obtain the Pareto solution to satisfy their subjective preference by giving a higher weight to the corresponding sub-objective. Figure 10 presents the area of each land-use zone determined in the aforementioned four scenarios.

It can be seen from Figure 10 that: (1) NHLPA in each scenario have the same area, because of the priority of being fixed, based on the general regulations for land-use zoning. The areas of BFPA and FLA are greater than 62,462.14 hm^2^ and 55,563.16 hm^2^, respectively, in each scenario, and the sum of the areas of CLV, CLT and IIMLA is less than 18,785.7 hm^2^, which indicated the efficiency of the constraint of area limitation of the various land-use zones; (2) Scenario M got the largest area of BFPA, CLT and IIMLA and the smallest area of FLA, CLV and TLA amongst the three scenarios. With the drive for spatial compactness, the large spatial clusters annexed the small clusters surrounding them to generate an optimal spatial pattern, simultaneously resulting in either a larger land-use zone or a smaller one; (3) Scenario N had the largest land-use zone of CLV and the second largest area of BFPA and GALA. This is because the spatial harmony between these land-use zones is higher than with the others; (4) The largest FLA was obtained in Scenario O because the forests can provide the greatest ESV. Meanwhile, Scenario O got the smallest BFPA and GALA. This suggests that the enforcement of the policy of ‘grain for green’ can improve the ESV of the land-use system; (5) Scenario L, assigning the same weight to each objective function, provided a compromise solution with a moderate value for each objective function (see Table 9) and area for each land-use zone.

**Figure 11:**
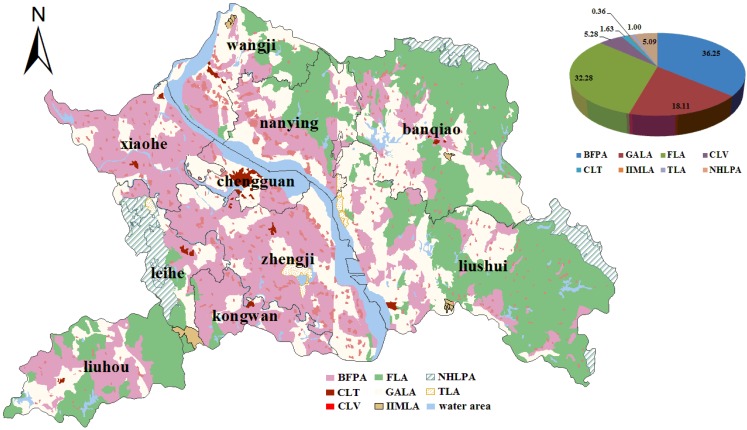
The spatial distribution and area proportions (%) of the eight land-use zones in Scenario L.

The global spatial distribution of the eight land-use zones in the four Scenarios of L, M, N, O are similar to each other, with only some microscopic differences. The reason for these disparities has been described above, with a vivid depiction in Figure 8 and Figure 9. As far as Scenario L is concerned, the quantity structure and spatial pattern of the land-use zones are presented in Figure 11.

The BFPA is distributed mainly in the towns of Xiaohe, Zhengji, Kongwan, Wangji and Nanying, and accounts for 36.25% of the total area, excluding the water areas. A total of 85.62% of the fifth level and 63.25% of the fourth level of agricultural land in the study area are assigned to BFPA. (The quality of agricultural land in Yicheng is classified into five levels: first level—the worst quality, second level, third level, fourth level, and fifth level-the highest quality.) This zone is marked off to hold its ability to continue feeding a growing population and meet the food security goals. High-quality land with high productivity in this zone should be prohibited from converting to non-agricultural uses. The conversion of good-quality land with moderate productivity to non-agricultural uses is permitted by law under some circumstances, usually after a planned period of 5–10 years [48]. GALA is observed mainly in the towns of Wangji, Nanying, Banqiao and Liushui, and is contiguous with BFPA. This zone primarily contains the land for gardens, aquaculture, *etc*., with high ecological value, and the rural roads, infrastructural facilities, water conservancy facilities, protective forest, *etc*., serve the farmland. In this region, the land consolidation project is encouraged to make the ‘dynamic balance of farmland’ policy materialize. FLA is concentrated to the east and southwest of Yicheng and mainly consists of fast-growing and high-yield broad-leaf forests. In view of the functions of forests, such as soil and water conservation, and ecological protection, the conversion of forest land is severely restricted, except for approval by the competent forestry authorities. The spatial clusters of CLV are evenly dispersed across the maps, the surroundings of which are mostly of BFPA and GALA, which annex some isolated rural settlements. At the same time, some farmland patches surrounding rural settlements are merged into them. CLT is marked off, based on actual land use for urban areas and towns, including some agricultural land, which can be used as the preparatory land for the future expansion of the city. Small villages, ‘hollow-village’ and ‘villages-in-the-city’ [49] within CLV and CLT should be amalgamated so as to upgrade the scale and organization of villages and towns. The IIMLA only covers around 0.34% of the territory of the city, and mainly comprises some land for the mining and chemical industries. The land that was destroyed in the process of industrial production and construction should be reclaimed for new use, especially cultivated land, if the land is suitable for agriculture. TLA is located in the vicinity of the region’s water areas, and NHLPA is distributed in the west, southeast and northeast corner of the city. In these regions, a small amount of agricultural land is permitted to improve the biodiversity and maintain the ecological balance, and the land for enterprises with industrial pollution is strictly prohibited.

### 4.2. Analysis of the Effect of Improvements for the Model

The focal point of this subsection is to analyze the effect of the adoption of the crossover and mutation operator on acquiring the optimal solutions. A comparative analysis of the optimal fitness values, convergence speed and run time is made to evaluate the performance of the MOPSO-CCM model and the basic particle swarm optimization with only a constriction factor (MOPSO-BC), in Scenarios I, J, K and L.

**Figure 12:**
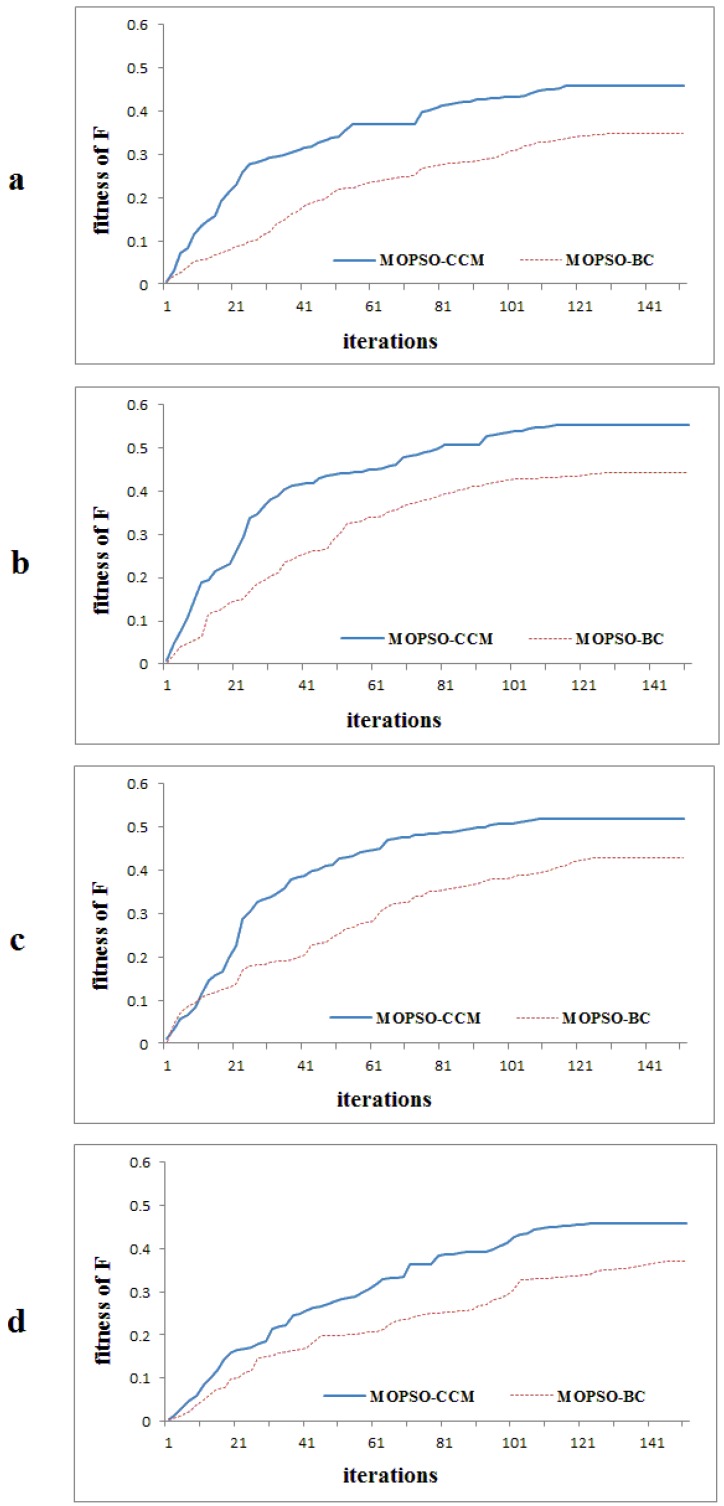
Optimization curves obtained by MOPSO-CCM and the MOPSO-BC model, respectively, with the four weighted scenarios: (**a**): I; (**b**): J; (**c**): K; (**d**): L.

It is observed in Table 10 that the optimal solution obtained by the MOPSO-CCM model exhibits the expected higher fitness value for each sub-objective in each scenario, with *f*_AD_ increasing by 15.6% to 17.48%, *f*_SC_ increasing by 14.61% to 16.89%, *f*_HD_ increasing by 9.6% to 14.18%, and *f*_EB_ increasing by 1.26% to 2.38%. Figure 12 clearly illustrates that the weighted sum fitness obtained by MOPSO-CCM in each generation is much higher than that of MOPSO-BC. At the same time, the convergence speed of MOPSO-CCM, with a sudden rise in its curve, is distinctly higher than that of MOPSO-BC, even if the initial normalized fitness value of MOPSO-CCM is lower than MOPSO-BC in Scenario K. The sudden rises in the curves of MOPSO-CCM in the later period of evolution show that the crossover and mutation operator helps the model to escape the local optimum and achieve a better solution. In summary, the convergence and exploration ability of the MOPSO-CCM model outperforms MOPSO-BC, which proves the validity of improvement for the PSO algorithm with land-use zoning. It is necessary, however, to note that MOPSO-CCM requires some computational cost to perform the operation of crossover and mutation on particles, based on a preset probability, so it is more time consuming than the MOPSO-BC model.

**Figure 13:**
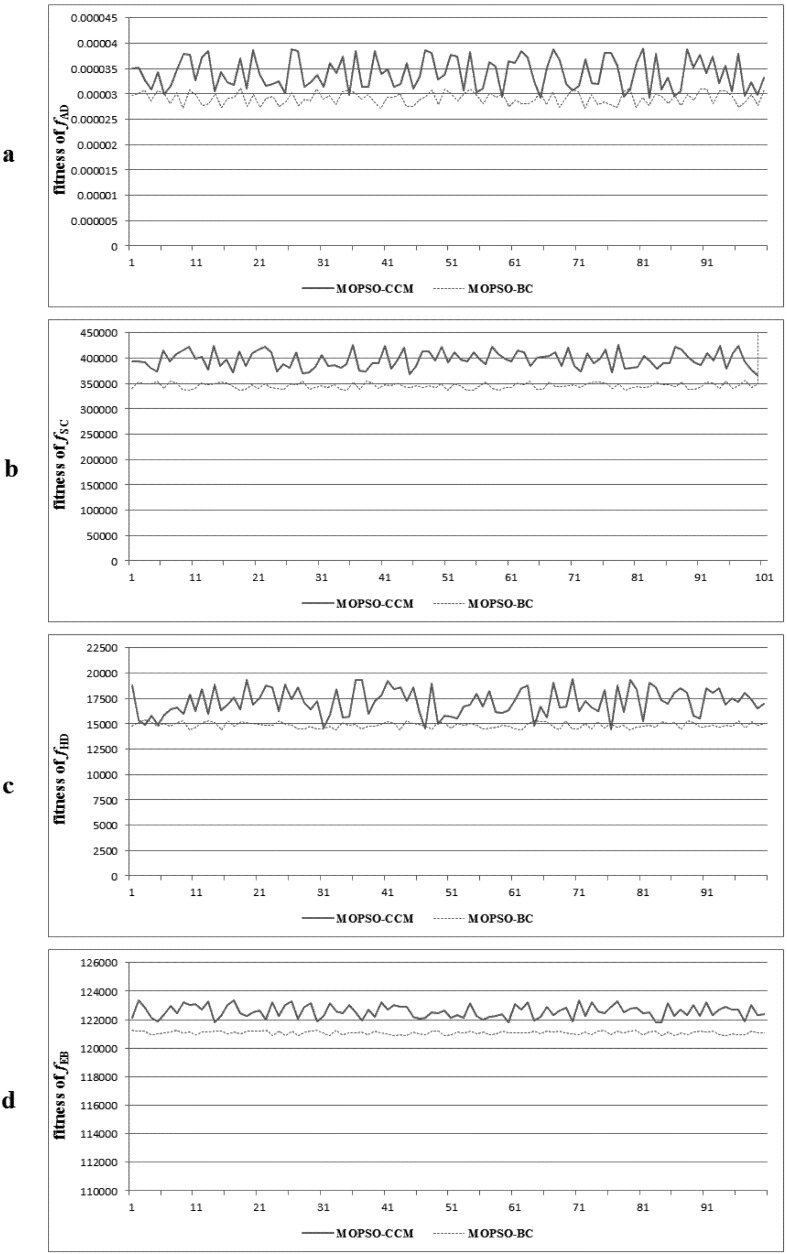
The fitness curves of the four sub-objectives obtained by MOPSO-CCM and MOPSO-BC with weighted scenario L: (**a**): *f*_AD_; (**b**): *f*_SC_; (**c**): *f*_HD_; (**d**): *f*_EB_.

A discussion of the stability of the improved PSO model is conducted next. We ran the MOPSO-CCM and MOPSO-BC model 100 times, respectively, with weighted Scenario L, and calculated the mean value and standard deviation of the four sub-objectives in each optimal solution (see Table 11). Although the mean value of MOPSO-CCM is obviously higher than MOPSO-BC, what further proves the improved exploration ability of MOPSO-CCM is that the standard deviation of MOPSO-BC is lower than MOPSO-CCM, which indicates that MOPSO-CCM is not as stable as MOPSO-BC. The optimal fitness values of each sub-objective are plotted in the curves. The curves of MOPSO-CCM in Figure 13 show a greater fluctuation than MOPSO-BC. The statistics table and graph reveal that the adoption of the crossover and mutation operator remarkably increases the probability of acquisition of an optimal solution by improving the diversity of the particle swarm, but also brings about some disturbance to the exploitation and exploration ability of the model.

**Table 10:** The results obtained by MOPSO-CCM and MOPSO-BC with the four weighted scenarios.

Scenario	Model	Fitness value				Convergence	Run time (h)
		*f*_AD_	*f*_SC_	*f*_HD_	*f*_EB_		
I	MOPSO-CCM	0.0000339	365,651	17,915.5	----	116	1.81
	MOPSO-BC	0.0000291	314,934	16,179.6	----	133	1.23
J	MOPSO-CCM	0.0000326	459,232	----	130,963.3	113	1.78
	MOPSO-BC	0.0000269	392,891	----	127,918.2	129	1.26
K	MOPSO-CCM	0.0000345	----	19,933.7	129,862.4	109	1.77
	MOPSO-BC	0.0000294	----	18,188.2	127,015.3	125	1.24
L	MOPSO-CCM	0.0000341	396,535	16,969.3	122,586.7	122	1.91
	MOPSO-BC	0.0000295	345,983	14,862.4	121,063.4	145	1.27

**Table 11:** The mean values and standard deviation of each sub-objective when the MOPSO-CCM and MOPSO- BC models are run 100 times.

Model	Mean value				Standard deviation			
	*f*_AD_	*f*_SC_	*f*_HD_	*f*_EB_	*f*_AD_	*f*_SC_	*f*_HD_	*f*_EB_
MOPSO-CCM	3.32E–05	409,341.2	17,852.36	121,834.6	3.05E–06	16,403.62	1,319.66	444
MOPSO-BC	2.92E–05	351,540.9	13,969.33	121,185.7	1.21E–06	5,619.41	281.29	116.56

## 5. Conclusions

The traditional approaches to the land-use zoning problem in China just take into consideration the characteristic attributes of the land units. In this article, the land-use zoning problem is treated as a MOP. Four objective functions were formulated, respectively, for attribute differences between land-use zones, spatial compactness, spatial harmony and the ecological benefits of land-use zones. A PSO-based model equipped with a crossover and mutation operator was constructed. The feasibility of the proposed approach in solving the land-use zoning problem was checked using a case study in Yicheng, China. The results obtained indicated that the integration of GIS and MOPSO-CCM is a promising and efficient approach for solving the land-use zoning problem. The inclusion of spatial compactness and spatial harmony in the objective functions allows the achievement of solutions with an optimal spatial pattern, and the inclusion of ecological benefits leads to a greater quantity of land-use zones with a high ESV. It is worth pointing out that the objective function can be extended in line with local conditions, such as the economic benefits for a county with a backward economy, spatial contiguity, *etc*. In this paper, a conventional weighted aggregation method is used for the MOPSO-CCM model to search for the Pareto-dominant solutions, the weakness of which is that an optimal solution can be obtained with only one run of the model. The next stage is to apply a dynamic weighted aggregation (DWA) technique to the MOPSO-CCM model to generate a series of Pareto-optimal solutions.

The incorporation of a crossover and mutation operator proved to be effective in improving the convergence and exploration ability, at the price of a relatively small increase in runtime and, simultaneously, a poorer stability than MOPSO-BC. As we know, the performance of PSO depends on a few different parameters [50]. Therefore, a further study will focus on choosing a group of parameters that will enable the MOPSO-CCM model to achieve a better performance with the land-use zoning problem.

## References

[1] Natoli S.J. (1971). Zoning and the development of urban land use patterns. Econ. Geogr..

[2] Rossi H.E. (2004). Optimal urban land use and zoning. Rev. Econ. Dyn..

[3] Mark J.H., Goldberg M.A. (1981). Land use controls: The case of zoning in the Vancouver Area. Real Estate Econ..

[4] McMillen D.P., McDonald J.F. (1999). Land use before zoning: The case of 1920’s Chicago. Reg. Sci. Urban Econ..

[5] Guo Y., Pu L.J., Zhao Y.Y., Hu X.T. (2007). Review and prospects of land use zoning research. Resour. Environ. Yangtze Basin.

[6] Fischel W.A., Boudewijn B., De Geest G. (2000). Zoning and Land Use Regulation. Encyclopaedia of Law and Economics.

[7] York A.M., Munroe Darla K. (2010). Urban encroachment, forest regrowth and land-use institutions: Does zoning matter?. Land Use Policy.

[8] Fleischmann A. (1989). Politics, administration, and local land-use regulation: Analyzing zoning as a policy proces. Public Adm. Rev..

[9] Wang J. (2001). Suggestion to the type of land use zones at the county level. China Land. Sci..

[10] Ou M.H. (2001). Discussion on the system of land use zoning. J. Nanjing Agric. Univ..

[11] Wang J. (2001). Land use zoning and regional sustainable land use. China Popul. Resour. Environ..

[12] Zhao R.Q., Huang X.J., Zhong T.Y., Xu H. (2010). Application of clustering analysis to land use zoning of coastal region in Jiangsu Province. Trans. Chin Soc. Agric. Eng..

[13] Meng L.N., Zheng X.Q., Zhao L., Deng J. (2011). Land-use functional regionalization based on niche-fitness model. Trans. Chin. Soc. Agric. Eng..

[14] Pan J.H., Liu Y., Shi P.J. (2011). Research on overall planning of land use division in Tianshui based on PCA and GIS. Soils.

[15] Wang J., Cheng Y., Liu K, Wang X.L., Huang X.J. (2003). The rational analysis of control on the usages of land. China Land Sci..

[16] Brookes C.J. (2001). A genetic algorithm for designing optimal patch configurations in GIS. Int. J. Geogr. Inf. Sci..

[17] Aerts J.C.J.H., Eisinger E., Heuvelink G.B.M., Stewart T.J. (2003). Using linear integer programming for multi-site land-use allocation. Geogr. Anal..

[18] Liu Y.F., Li X.L., Gong H.B. (2005). Optimization for land use structure based on genetic algorithms. Geomat. Inf. Sci. Wuhan. Univ..

[19] Sante R.I., Boullon M.M., Crecente M.R., Miranda B.D. (2008). Algorithm based on simulated annealing for land-use allocation. Comput. Geosci..

[20] Sadeghi S.H.R., Jalili K., Nikkami D. (2009). Land use optimization in watershed scale. Land Use Policy.

[21] Gao Q.Z., Kang M.Y., Xu H.M., Jiang Y., Yang J. (2010). Optimization of land use structure and spatial pattern for the semi-arid loess hilly-gully region in China. Catena.

[22] Eldrandaly K. (2010). A GEP-based spatial decision support system for multisite land use allocation. Appl. Soft Comput..

[23] Zhang H.H., Zheng Y.N., Tan R., Liu H.M. (2011). A model for regional land use optimization allocation based on multi-agent system and its application. Acta Geogr. Sin..

[24] Stewart T.J., Janssen R., Van H.M. (2004). A genetic algorithm approach to multi-objective land use planning. Comput. Oper. Res..

[25] Duh J.D., Brown D.G. (2007). Knowledge-informed Pareto simulated annealing for multi-objective spatial allocation. Comput. Environ. Urban Syst..

[26] Kennedy J., Eberhart R.C. Particle Swarm Optimization. Proceedings of the 1995 IEEE International Conference on Neural Networks.

[27] Wang Y., Li B., Weise T., Wang J.Y., Yuan B., Tian Q.J. (2011). Self-adaptive learning based particle swarm optimization. Inf. Sci..

[28] Nickabadi A., Ebadzadeh M.M., Safabakhsh R. (2011). A novel particle swarm optimization algorithm with adaptive inertia weight. Appl. Soft Comput..

[29] Suresh S., Sujit P.B., Rao A.K. (2007). Particle swarm optimization approach for multi-objective composite box-beam design. Compos. Struct..

[30] Okamoto T.S., Aiyoshi E. (2008). Global optimization with the PSO coupling-type discrete gradient chaos model. Electr. Eng. Jpn..

[31] Tsafarakis S., Marinakis Y., Matsatsinis N. (2011). Particle swarm optimization for optimal product line design. Int. J. Res. Mark..

[32] Beghi A., Cecchinato L., Cosi G., Rampazzo M. (2012). A PSO-based algorithm for optimal multiple chiller systems operation. Appl. Therm. Eng..

[33] Clerc M., Kennedy J. (2002). The particle swarm-explosion, stability, and convergence in a multidimensional complex space. IEEE Trans. Evol. Comput..

[34] Higashi N., Iba H. Particle Swarm Optimization with Gaussian Mutation. Proceedings of IEEE Swarm Intelligence Symposium.

[35] Yong Y., Zhang H., Wang X.R., Schubert U. (2010). Urban land-use zoning based on ecological evaluation for large conurbations in less developed regions: Case study in Foshan, China. J. Urban Plan. Dev. ASCE.

[36] Kong X.S., Liu Y.F., Tan C.F. (2009). Correlation analysis of the changes of land use structure and land use efficiency: A case study of Jiayu County in Hubei Province. Resour. Sci..

[37] Xie G.D., Lu C.X., Leng Y.F., Li S.C. (2003). Ecological assets valuation of the Tibetan Plateau. J. Nat. Resour..

[38] Costanza R., d’Arge R., deGroot R., Farber S., Grasso M., Hannon B., Limburg K., Naeem S., OʼNeill R.V., Paruelo J. (1997). The value of the world’s ecosystem services and natural capital. Nature.

[39] Sang L.L., Zhang C., Yang J.Y., Zhu D.H., Yun W.J. (2011). Simulation of land use spatial pattern of towns and villages based on CA-Markov model. Math. Comput. Model..

[40] Verburg P.H., de Koning G.H.J., Kok K., Veldkamp A., Bouma J. (1999). A spatial explicit allocation procedure for modelling the pattern of land use change based upon actual land use. Ecol. Model..

[41] Cheng Y., Wang J., Meng F.H. (2003). Reference. Research of Space Control of Land Use Zoning.

[42] Nickabadi A., Ebadzadeh M.M., Safabakhsh R. (2011). A novel particle swarm optimization algorithm with adaptive inertia weight. Appl. Soft Comput..

[43] Shi Y.H., Eberhart R.C. Parameter Selection in Particle Swarm Optimization. Proceedings of Evolutionary Programming VII 7th International Conference, EP98.

[44] Eberhart R.C., Shi Y. Comparing Inertia Weights and Constriction Factors in Particle Swarm Optimization. Proceedings of 2000 Congress on Evolutionary Computing (CEC2000).

[45] Clerc M. The Swarm and the Queen: Towards a Deterministic and Adaptive Particle Swarm Optimization. Proceedings of ICEC.

[46] Trelea I.C. (2003). The particle swarm optimization algorithm: Convergence analysis and parameter selection. Inf. Proc. Lett..

[47] Carlisle A., Dozier G. An Off-The-Shelf PSO. Proceedings of the Workshop on Particle Swarm Optimization. Indianapolis, Purdue School of Engineering and Technology, IUPUI.

[48] Lichtenberg E., Ding C.R. (2008). Assessing farmland protection policy in China. Land Use Policy.

[49] Long H.L., Liu Y.R., Liu Y.S., Woods M., Zou J. (2012). Accelerated restructuring in rural China fueled by ‘increasing *vs*. decreasing balance’ land-use policy for dealing with hollowed villages. Land Use Policy.

[50] Parsopoulos K.E., Vrahatis M.N. (2007). Parameter selection and adaptation in unified particle swarm optimization. Math. Comput. Model..

